# Comment on “Risk factors associated with chronic cutaneous graft-versus-host disease following hematopoietic stem cell transplantation: a pediatric cohort” – Reply^☆^

**DOI:** 10.1016/j.abd.2026.501459

**Published:** 2026-09-01

**Authors:** Daniela Carvajal, Teresa Dossi, Camila Downey, Daniela Krämer, Natalia González, Javier Fernández, Paula Muñoz

**Affiliations:** a Department of Pediatric Dermatology, Hospital de Niños Luis Calvo Mackenna, Santiago, Chile; b Department of Oncology, Hospital de Niños Luis Calvo Mackenna, Santiago, Chile; c Clínica Santa María, Santiago, Chile; d Department of Dermatology, Universidad de Los Andes, Santiago, Chile

Dear Editor,

We appreciate the insightful comments regarding our article. Such technical discussions are essential for refining our understanding of chronic cutaneous Graft-Versus-Host Disease (ccGVHD) in the pediatric population. We would like to address the points raised with additional statistical insights that further validate our original findings.

## Structural collinearity: graft source vs. donor type

The observed association between these variables is a reflection of the structural collinearity inherent to the clinical protocols and historical resource availability in Chile during the study period (2007–2017). Prior to the establishment of international donor registries in the country (such as DKMS, which began operations in Chile after 2017), access to unrelated bone marrow (BM) donors was severely limited. Consequently, Umbilical Cord Blood (UCB) became the primary source for unrelated transplants.

Clinically, our institutional protocol prioritizes BM over Peripheral Blood Stem Cells (PBSC) in children to minimize the risk of GVHD and graft failure. This strategy is consistent with recent evidence indicating that PBSC grafts are associated with a higher risk of chronic GVHD compared to BM or UCB.^1^ Graft source was prioritized in our final model because it reflects the actual cellular inoculum and mature T-cell burden ‒ providing a more direct pathophysiological surrogate for ccGVHD than the donor-recipient genetic relationship alone. To ensure the robustness of this finding, we performed a sensitivity analysis using a multivariable model adjusted for age, Total-Body Irradiation (TBI), and acute cutaneous GVHD (acGVHD) history. In this model, BM maintained a highly significant independent impact (HR = 4.98; 95% CI 1.85–13.44; p = 0.001), confirming that the graft source is the primary driver of risk in our cohort regardless of other clinical variables.

## Acute cutaneous GVHD as a baseline predictor

Regarding the suggestion to treat acGVHD as a time-dependent covariate, we acknowledge its statistical merit. However, our objective was to identify baseline risk factors that are actionable from “day zero” of the Hematopoietic Stem Cell Transplantation (HSCT).

To address the hierarchy of these events, we evaluated the variables within the same adjusted multivariable model described in the previous point. We observed that the significance of acGVHD (p = 0.041 in the univariate analysis) was attenuated to p = 0.141 when the graft source was included. In contrast, Bone Marrow (BM) retained its full statistical power (p = 0.001).

This statistical shift suggests that the graft source is the primary “upstream” determinant in our population; the risk of ccGVHD appears largely predetermined by the cell source received. Since this information is available at the time of transplantation, it remains a more robust and clinically actionable predictor for early dermatological surveillance than the subsequent onset of acGVHD.

## TBI and diagnostic independence

The hypothesis that TBI serves as a proxy for disease severity is not supported by our clinical or statistical data. First, it is important to note that the underlying diagnosis (acute myeloid leukemia, Acute Lymphoblastic Leukemia [ALL], or others) was not a significant predictor of ccGVHD in our univariate analysis (p = 0.254 and p = 0.364, respectively). According to standard statistical parsimony, the variable “Diagnosis” was therefore not included in our final multivariable model.

Second, although TBI use is clinically linked to ALL in our center (94% of irradiated patients in our cohort had ALL), TBI remained a robust and highly significant independent risk factor in our original multivariable model (p = 0.003), even when adjusted for other significant predictors such as sex and graft source. These findings reinforce the conclusion that TBI confers a direct biological risk independently of the primary malignancy, consistent with findings from other studies. A Spanish study of 50 children with acute and chronic cutaneous GVHD published 2023, showed that children with ccGVHD had a significantly higher proportion of TBI versus patients without ccGVHD (66% vs. 33%).^2^

## Conclusion

Our additional analyses confirm that the published findings are robust and clinically relevant. Reporting the reality of a Latin American center in the pre-international registry era provides unique value to the global literature. We reaffirm our recommendation for strict dermatological surveillance during the first 15-months post HSCT, especially for those receiving BM or TBI-based conditioning. Finally, we thank the authors of the correspondence for providing the opportunity to further discuss these important clinical and statistical considerations.

## ORCID ID

Daniela Carvajal: 0000-0003-1723-9002

Teresa Dossi: 0009-0008-6838-2881

Camila Downey: 0000-0002-1624-1170

Daniela Krämer: 0000-0003-3022-3187

Natalia González: 0009-0000-8903-3770

Javier Fernández: 0000-0003-1723-9002

Paula Muñoz: 0000-0003-2676-7464

## Research data availability

Does not apply.

## Financial support

None declared.

## Authors’ contributions

Daniela Carvajal: Writing of the manuscript or critical review of important intellectual content; critical review of the literature; final approval of the final version of the manuscript.

Teresa Dossi: Data collection, analysis and interpretation; critical review of the literature; final approval of the final version of the manuscript.

Camila Downey: Data collection, analysis and interpretation; critical review of the literature; final approval of the final version of the manuscript.

Daniela Krämer: Data collection, analysis and interpretation; critical review of the literature; final approval of the final version of the manuscript.

Natalia González: Data collection, analysis and interpretation; critical review of the literature; final approval of the final version of the manuscript.

Javier Fernández: Data collection, or analysis and interpretation of data; statistical analysis; final approval of the final version of the manuscript.

Paula Muñoz: The study concept and design; data collection, or analysis and interpretation of data; data collection, analysis and interpretation; effective participation in the research guidance; critical review of the literature; and final approval of the final version of the manuscript.

## Conflicts of interest

None declared.
